# Generation of minipigs with targeted transgene insertion by recombinase-mediated cassette exchange (RMCE) and somatic cell nuclear transfer (SCNT)

**DOI:** 10.1007/s11248-012-9671-6

**Published:** 2012-10-31

**Authors:** Jannik Ejnar Jakobsen, Marianne G. Johansen, Mette Schmidt, Frederik Dagnæs-Hansen, Karen Dam, Anders Gunnarsson, Ying Liu, Peter M. Kragh, Rong Li, Ida E. Holm, Henrik Callesen, Jacob Giehm Mikkelsen, Anders Lade Nielsen, Arne Lund Jørgensen

**Affiliations:** 1 Department of Biomedicine, Aarhus University, Wilhelm Meyers Alle′ 4, building 1240, 8000 Aarhus C, Denmark; 2 Department of Animal Science, Faculty of Science and Technology, Aarhus University, 8830 Tjele, Denmark; 3 Department of Veterinary Reproduction and Obstetrics, Faculty of Life Sciences, University of Copenhagen, 1870 Frederiksberg C, Denmark; 4 Laboratory for Experimental Neuropathology, Department of Pathology, Randers Hospital, 8900 Randers, Denmark

**Keywords:** RMCE, Sleeping Beauty, SCNT, Targeted transgenesis, Transgenic pigs, Disease models

## Abstract

Targeted transgenesis using site-specific recombinases is an attractive method to create genetically modified animals as it allows for integration of the transgene in a pre-selected transcriptionally active genomic site. Here we describe the application of recombinase-mediated cassette exchange (RMCE) in cells from a Göttingen minipig with four RMCE acceptor loci, each containing a green fluorescence protein (GFP) marker gene driven by a human UbiC promoter. The four RMCE acceptor loci segregated independent of each other, and expression profiles could be determined in various tissues. Using minicircles in RMCE in fibroblasts with all four acceptor loci and followed by SCNT, we produced piglets with a single copy of a transgene incorporated into one of the transcriptionally active acceptor loci. The transgene, consisting of a cDNA of the Alzheimer’s disease-causing gene *PSEN1M146I* driven by an enhanced human UbiC promoter, had an expression profile in various tissues similar to that of the GFP marker gene. The results show that RMCE can be done in a pre-selected transcriptionally active acceptor locus for targeted transgenesis in pigs.

**Electronic supplementary material:**

The online version of this article (doi:10.1007/s11248-012-9671-6) contains supplementary material, which is available to authorized users.

## Introduction

Genetically modified animals are widely used in studies of human diseases with a genetic etiology. To this end the mouse has played and plays an important role in translational biomedical research, but for a number of human diseases, the mouse is not an eligible model. Due to higher resemblance to humans with regard to size, longevity, anatomy, and physiology the pig may represent a prime candidate for creating animal models of human diseases that have failed in the mouse [for review see(Whyte and Prather 2011)]. Cystic fibrosis is a prominent example of a disease which is successfully replicated in the pig, whereas mouse models have not fully mimicked the pathology (Rogers et al. 2008a, b; Ostedgaard et al. 2011). It is generally accepted that genetic manipulation of the genome of an animal model should be as restricted as possible and require as few animals as possible. These efforts may limit side effects such as increased abortion and newborn mortality rates and malformations as they could be caused by uncontrolled integration of transgenes into the genome with the risk of disrupting the animal’s genes. Using random integration techniques also raises concern about transgene copy number, integration of incomplete transgenes, and transgene incorporation into or near transcriptionally inactive heterochromatin. Targeted transgenesis may be a way to circumvent these concerns. One method to produce animals with targeted insertion is homologous recombination (HR) in embryonic stem cells (ESCs) that are subsequently injected into blastocysts to produce chimeric offspring (Doetschman et al. 1987; Thompson et al. 1989; Capecchi 1989). However, ESCs have not yet been isolated from the pig (Brevini et al. 2010a, b). Site specific recombinases (SSRs) are available for efficient targeted transgene insertion into the genome. Especially, the use of the Cre and Flp SSRs in recombinase-mediated cassette exchange (RMCE) has proven efficient for transgene targeting in the mouse genome (Osterwalder et al. 2010; Schnutgen et al. 2005; Cobellis et al. 2005; Schebelle et al. 2010). The Sleeping Beauty (SB) DNA transposon system is well established in the mouse (Dupuy et al. 2001, 2005; Carlson et al. 2003, 2005, 2011b; Mates et al. 2009; Kitada et al. 2007; Geurts et al. 2006) and has been used to transfer transgenes into the genome of porcine cells (Clark et al. 2007; Jakobsen et al. 2011a, b; Carlson et al. 2011a). Some of these cells have subsequently been used for SCNT to produce piglets (Carlson et al. 2011a; Jakobsen et al. 2011a). Recently, Garrels et al. (2011) microinjected the components of the SB system into porcine zygotes to generate transgenic pigs. They used fibroblasts from a fetus with a single transposon integration for targeted transgenesis by RMCE followed by SCNT and produced fetuses that expressed red fluorescent protein.

Here we present a porcine system for targeted transgenesis using minicircle DNA in fibroblasts from a healthy minipig that harbors four SB transposons acting as acceptor loci for RMCE. Subsequent somatic cell nuclear transfer (SCNT) and embryo transfer to recipient Danish landrace sows resulted in live born and healthy minipiglets carrying in their genome a target-inserted and transcriptionally active transgene. No random integrations were found in the genomes of the RMCE-generated piglets. With the acceptor loci segregating independently we could determine the transcriptional activity of the acceptor loci.

## Results

### Generation of pigs containing RMCE acceptor loci

**Fig. 1:**
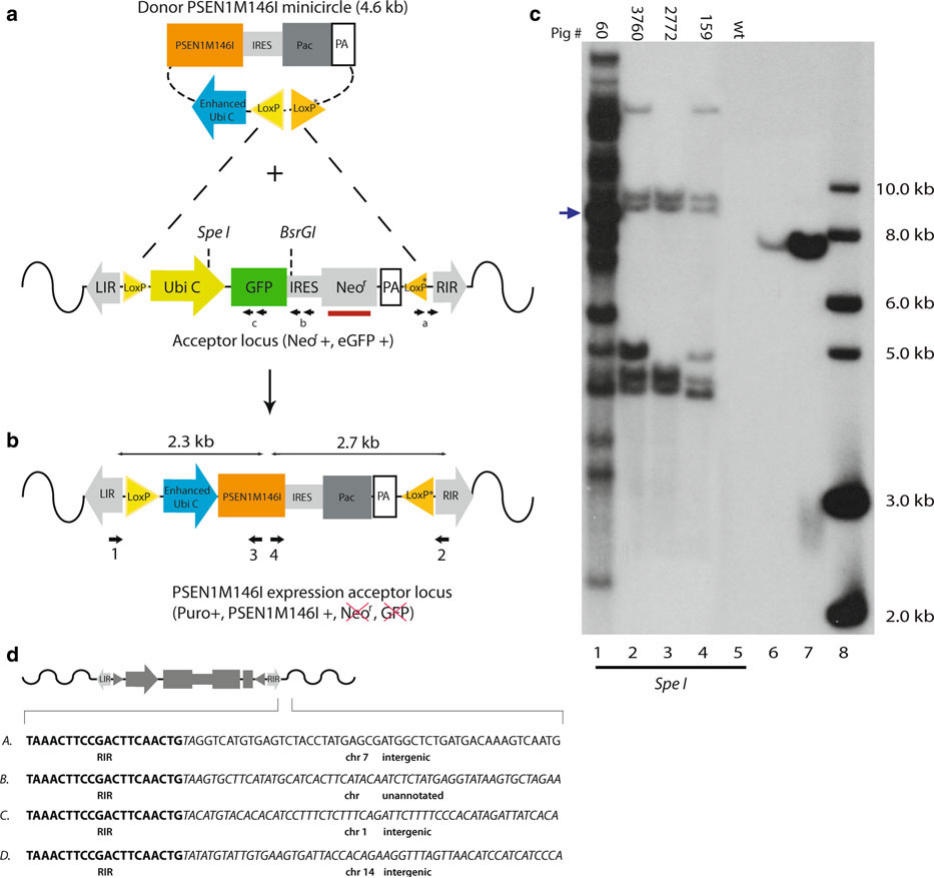
Establishment of Sleeping Beauty DNA transposon transgenic pigs for RMCE. **a** Schematic representation of Cre mediated RMCE in the pSBT/floxedUbi-GIN acceptor locus. The acceptor locus consists of a Sleeping Beauty DNA transposon with the GFP gene (*green rectangle*) linked to neomycin resistant gene (*Neo* ^*r*^, *grey rectangle*) through an internal ribosomal entry site (IRES, *light grey rectangle*). This unit is controlled by the humane ubiquitin C promoter (Ubi C, *light green arrow*) and a SV40 polyadenylation signal (PA). Two incompatible loxP sites (*yellow triangles*; *Asterisk* indicates mutated loxP site) flank the cassette for RMCE. The transposon unit is demarcated by LIR and RIR (*grey arrows*). The RMCE donor minicircle is composed of a CMV enhanced Ubi C promoter (*blue arrow*) controlling the *PSEN1M146I* gene (*orange rectangle*) linked to the puromycin resistance gene, *Pac* (*grey rectangle*), through an IRES element (*small grey rectangle*). **b** Schematic representation of the acceptor locus after RMCE. Primers to verify RMCE are marked with small *black arrows* and the corresponding lengths of the PCR products marked by *thin arrows*. **c** Southern blot analysis of genomic DNA from SBT/floxedUbi-GIN–transgenic pigs and wt pig digested with *SpeI* (lanes 1–5). A 670-bp Neo^r^ fragment was used as probe (*red rectangle* in **a**). Pig identification numbers are shown above lanes. *Lanes 6 and 7* include *BamHI*-digested pSBT/floxedUBi-GIN representing DNA amounts equivalent to one and twenty copies, respectively. *Lane 8*, DNA ladder. The *blue arrow marks* a putative concatemer **d** Junction site sequences identified by LDI-PCR in pig #2772 harboring four copies of SBT/floxedUBi-GIN (**a**, **b**, **c**, **d**). (Color figure online)

The pSBT/floxedUbi-GIN plasmid was co-transfected with a plasmid encoding the HSB3 transposase (Jakobsen et al. 2011a) into fibroblasts isolated from a male Göttingen minipig. SBT/floxedUbi-GIN-transgenic fibroblasts were used for handmade cloning which is a variant of SCNT (Du et al. 2007), to produce piglets carrying the SBT/floxedUBi-GIN transposon, from which cells could be subjected to RMCE (Fig. 1a, b). Three transgenic pigs (F0) were born without any visible abnormalities, and their clonal origin was confirmed by Southern blotting which showed identical transgene hybridization patterns (data not shown). Multiple integration sites were observed in these F0 pigs (Fig. 1c lane 1), and some of the integrations appeared to be in the form of concatemers (heavy band marked by blue arrow in Fig. 1c, lane 1). It was therefore decided to produce F1 piglets with a lower number of integrations by breeding a F0 pig (#60) with wt minipig sows. Twenty-six F1 piglets were subsequently produced of which seven were estimated by qPCR on genomic DNA to have an acceptor locus copy number below ten and with no concatemers (supplementary Fig. S1a). Three of these seven F1 pigs with highest GFP mRNA levels (supplementary Fig. S1b) were used for Southern blotting to analyze the exact acceptor loci copy number (Fig. 1c). Four integrations were observed for one male pig, #2772 (Fig. 1c, lane 3), whereas the remaining two pigs, #3760 and #159, had six integrations (Fig. 1c, lane 2 and 4, respectively). LDI-PCR was used to map the genomic location of the four RMCE acceptor loci of pig #2772 and revealed that all acceptor loci were integrated as SB transposons, evident from the sequences showing that each transgene cassette was demarcated by inverted repeats flanked by TA-dinucleotides (Fig. 1d). Furthermore, three of the RMCE acceptor loci (A,C,D) were mapped to chromosomes 7, 1, 14, respectively, while locus B resided in unannotated sequences (Fig. 1d). With three, possibly all four loci, unlinked it was possible to produce F2 piglets with only a single RMCE acceptor locus (see later).

### Using pig #2772-derived fibroblasts for RMCE integration of PSEN1M146I minicircles

**Fig. 2:**
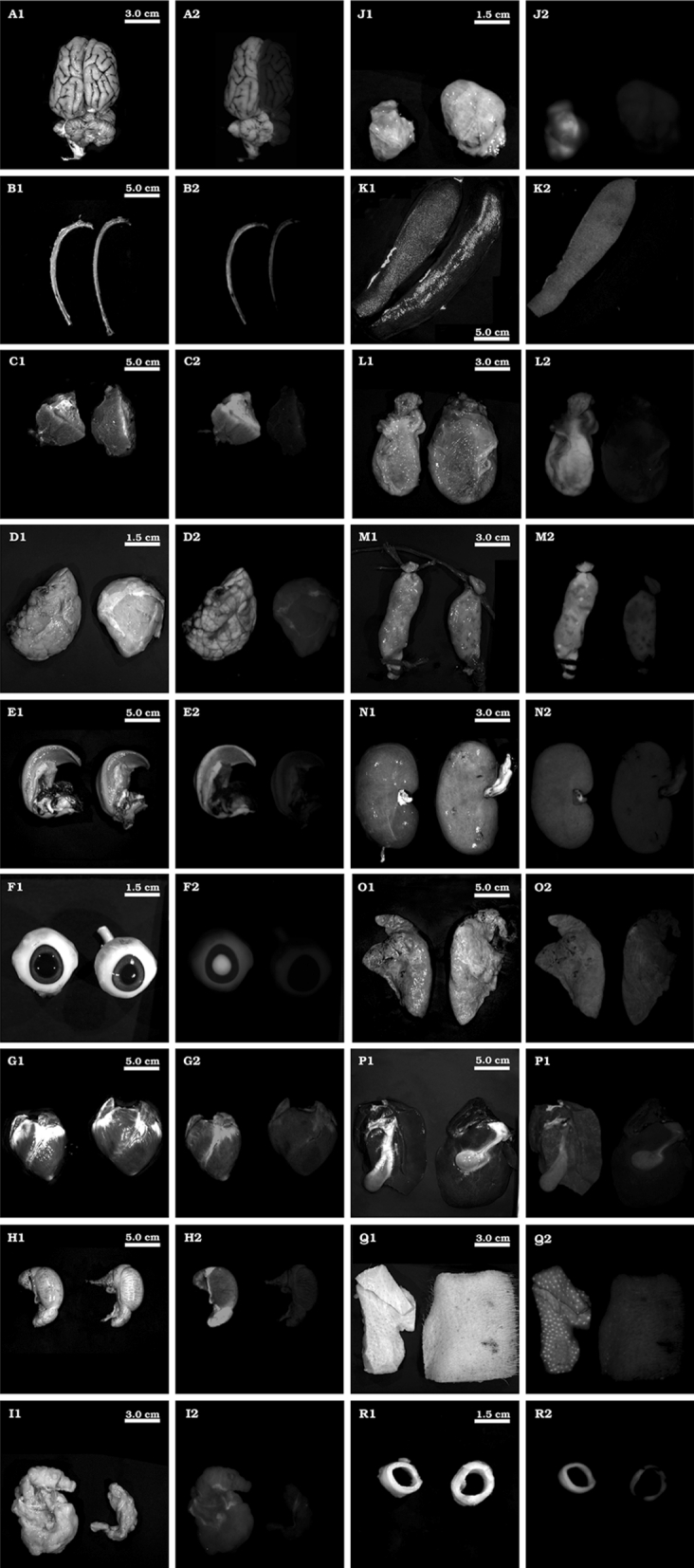
Systemic GFP expression in pig #2772. The *left* and *right* side of each picture show organ/tissue from pig #2772 and a wt pig, respectively: Brain (A), Rib bone (B), Skeletal muscle (C), Salivary gland (D), Tongue (E), Eye (F), Heart (G), Testis (H), Fat (I), Lymph node (J), Spleen (K), Bladder (L), Colon (M), Kidney (N), Lung (O), Liver (P), Skin (Q) and Aorta (R). Diagrams A1 through R1 show samples under normal light displayed in *black and white*. A2 through R2 show samples under *blue light* excitation (480 nm). (Color figure online)

After F1 pig #2772 had been used for breeding it was sacrificed and we examined eighteen organs/tissues all of which appeared macroscopically normal and exhibited GFP expression (Fig. 2). In addition, all the fibroblasts isolated from pig #2772 showed uniform green fluorescence (Supplementary Fig. S1c). Fibroblasts were used for RMCE as schematically depicted in Fig. 1a and b. Colonies derived from fibroblasts subjected to RMCE and puromycin selection were analyzed for cassette exchange with *PSEN1M146I* minicircles by PCR using primers 1 and 3 (Fig. 1b and Supplementary, Fig. S2c). Furthermore, expression of *PSEN1M146I* and the Cre recombinase was assessed by RT-PCR (Supplementary, Fig. S2a–b). Out of 19 harvested colonies, 18 could be expanded to give enough cells for DNA and RNA analysis. All 18 colonies had *PSEN1M146I* integrated into an RMCE acceptor locus and 16 showed expression of *PSEN1M146I*. Only one cell colony showed continuous expression of Cre over a time period of 3 weeks. Colonies 10, 15, and 16 were subsequently picked for SCNT as they displayed healthy fibroblast morphology and had a relatively high expression level of *PSEN1M146I*-IRES-*Pac* similar to the expression level of the *RPL4* control gene (data not shown). Furthermore, PCR on DNA from colonies 10, 15, and 16 using primers 4 and 2 (Fig. 1b) verified the correct RMCE (Supplementary, Fig. S2d). Finally, in all three colonies, the bicistronic *PSEN1M146I*-IRES-*Pac* mRNA was amplified by 3′ race and the correct sequence verified (data not shown).

### Piglets with targeted transgene insertion produced by RMCE and SCNT

**Fig. 3:**
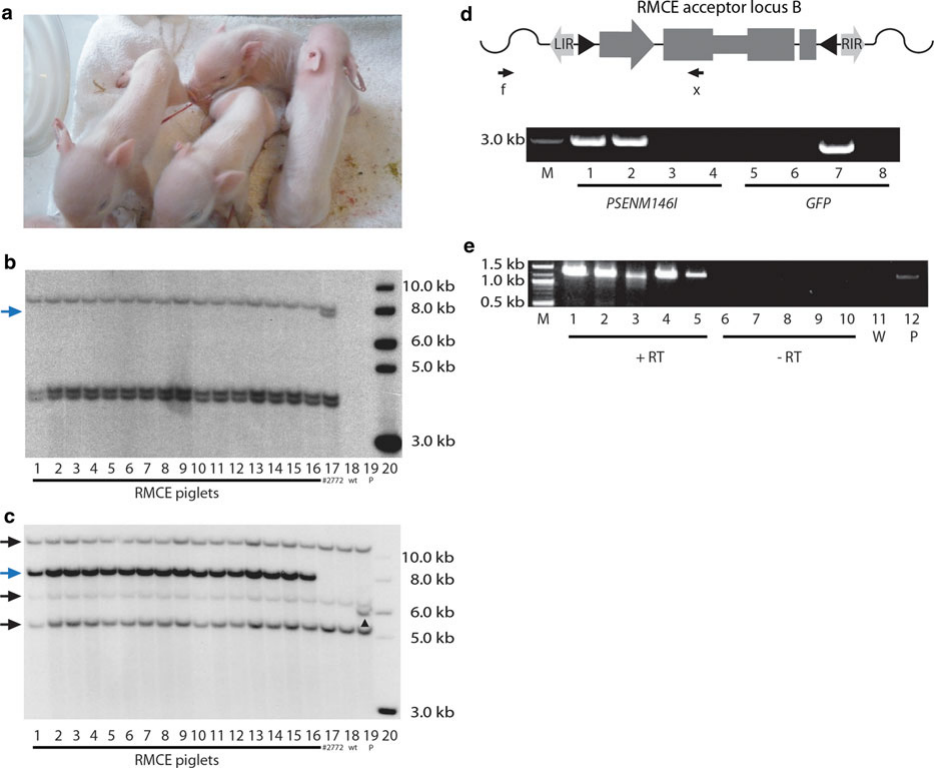
Generation of live born *PSEN1M146I* RMCE piglets. **a** Piglets generated by RMCE and SCNT. Four of 20 live born piglets are shown. **b** Southern blot analysis of genomic DNA isolated from 16 RMCE piglets and pig #2772 digested with *SpeI*. A 670-bp Neo^r^ fragment was used as probe. *Lanes 1–16* represent 16 RMCE piglets, *lane 17* pig #2772, *lane 18* wt pig, *lane 19* wt pig DNA mixed with *PSEN1M146I* plasmid DNA, and *lane 20* molecular weight marker. The *blue arrow* marks the band in pig #2772 absent in RMCE piglets. **c** Southern blot analyses as in **b** except for the use of a *PSEN1M146I* probe. The *blue arrow* marks the *PSEN1M146I* transgene present in RMCE piglets. Three other bands present in the wt pig and pig #2772 are marked with *black arrows* (endogenous *PSEN1*). Positive control band (*lane 19*) is marked with a *black triangle*. **d** *Top panel* Schematic drawing of the RMCE targeted acceptor locus B. *Black arrows* indicate positions of primers used to reveal RMCE. **f** *Arrow* marks the forward genomic primer upstream of LIR and x marks the reverse primer specific of either *PSEN1M146I* or GFP. *Lower panel* PCR on genomic DNA from two RMCE piglets (lanes 1, 2, 5, and 6) and pig #2772 (*lanes 3 and 7*). *Lanes 4 and 8* are water controls. **f** Primer was used with primer x, *PSEN1M146I* or GFP, in *lanes 1–4* and 5–8, respectively. M is a 1 kb ladder. **e** Expression of bi-cistronic *PSEN1M146I*-IRES-*Pac* mRNA in fibroblasts of five RMCE piglets. *Lanes 1–5* PCR on cDNA synthesized from fibroblast RNA, *lanes 6–10* control PCR on –RT templates, *lane 11* water control (W), and lane 12 positive control (P). M, 0.1 kb ladder

To reduce the number of passages necessary to have enough cells, colonies 10, 15, and 16 were pooled and used for SCNT, and this resulted in the birth of 21 piglets (Fig. 3a). One of the piglets was stillborn, one died shortly after birth, one pig had arthrogryposis, and one pig had macroglossia. Three piglets, including the stillborn and the piglet that died shortly after birth, had an average birth weight of 0.282 kg, whereas the remaining piglets had an average birth weight of 0.578 kg. We have previously described similar observations in cloned pigs (Schmidt et al. 2010, 2011). A Southern blot using *SpeI*-digested DNA isolated from 16 RMCE piglets and using the same Neo^r^ probe as for the Southern blot presented in Fig. 1c was performed to reveal which RMCE acceptor loci had been targeted (Fig. 3b, blue arrow). The Southern blot showed that the second largest band in pig #2772 was missing in the RMCE piglets (Fig. 3b, compare lanes 1–16 with lane 17). This band corresponds to the RMCE acceptor locus at transposon integration site B (Fig. 1d). The membrane used for the Southern blot was stripped and probed with a *PSEN1M146I* probe to reveal any *PSEN1M146I* integrations (Fig. 3c). An 8 kb band (Fig. 3c, blue arrow) was visualized in the RMCE piglets but not in pig #2772 or the wt pig (Fig. 3c compare lanes 1–16 with lanes 17 and 18, respectively), indicating that the Ubi-*GFP*-IRES-*Neo* ^*r*^ gene cassette had been replaced by the enhanced-Ubi-*PSEN1M146I*-IRES-*Pac* cassette. Three other bands were observed also in the wt pig and pig #2772. Alignment of the sequences of the *PSEN1M146I* probe and endogenous *PSEN1* revealed that these bands represent *SpeI* (digestion enzyme used for the Southern blotting) digested fragments of endogenous *PSEN1*. No other bands were visualized indicating that no random integration of *PSEN1M146I* had occurred (Fig. 3c). The identical Southern blot patterns seen in the 16 pigs (lanes 1–16) indicated that these pigs originated from only one of the three pooled cell colonies (see Discussion). We performed PCRs with a genomic primer outside the left inverted repeat of the transposon and a *PSEN1M146I*- or GFP-specific primer to verify that RMCE had indeed taken place at the indicated acceptor locus (Fig. 3d). A band was only observed in the RMCE pigs and not in pig #2772 when using the *PSEN1M146I*-specific primer combined with the genomic primer, whereas a band could only be observed for pig #2772 and not the RMCE pigs when using the GFP specific primer (Fig. 3d). Furthermore, all the RMCE piglets have been targeted at the RMCE acceptor locus B (Supplementary, Fig. S3d) and not in any of the remaining acceptor loci (Supplementary, Fig. S3a–c). However, we have obtained sequence information that shows that these loci could be targeted in fibroblasts from pig #2772 (data not shown). Expression of the *PSEN1M146I*-IRES-*Pac* cassette from its position within the acceptor locus B was confirmed by RT-PCR performed on RNA isolated from fibroblasts of RMCE piglets (Fig. 3e). Also, fibroblasts isolated from the RMCE piglets showed resistance to puromycin, indicating that the cistron was efficiently translated (data not shown).

### Expression profile of the individual RMCE loci in F2 progeny of F1 pig #2772

**Fig. 4:**
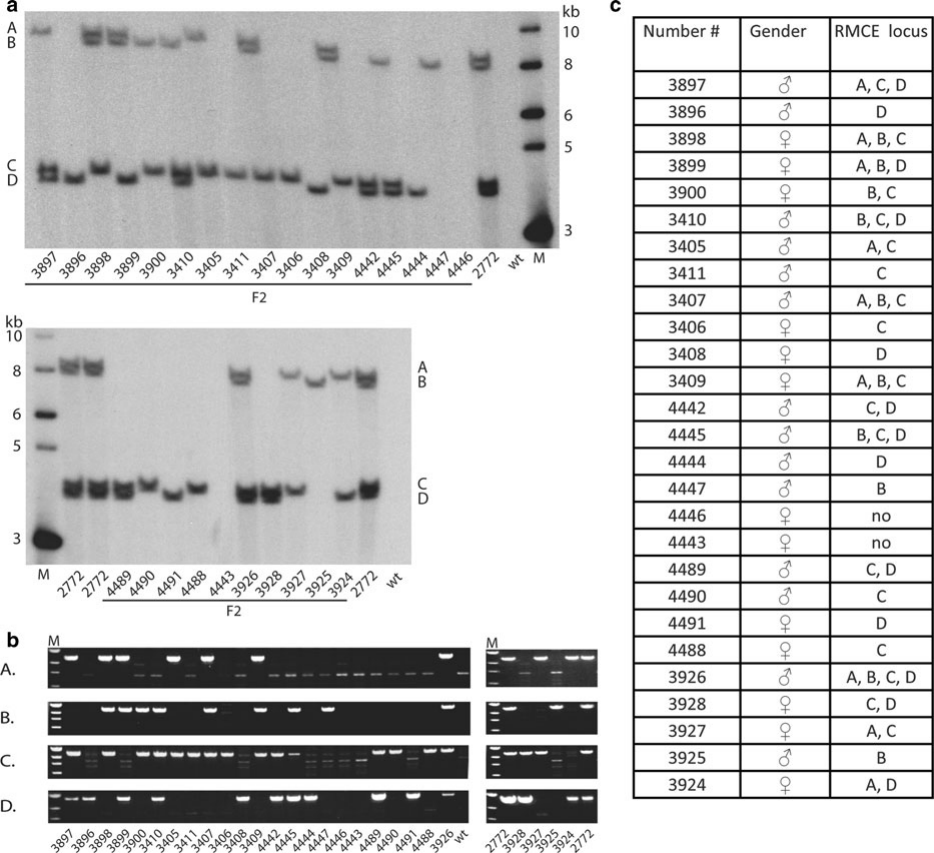
Independent segregation of RMCE acceptor loci in F2 progeny from pig #2772. Pig #2772 was bred with a wt pig to create 27 piglets with various RMCE acceptor loci. **a** The acceptor loci were revealed by Southern blot using the same condition as in Fig. 1c. The F2 pigs number listed beneath each lane. DNA ladder marked M. **b** The RMCE acceptor loci integration site were confirmed by PCR using a GFP primer and a genomic primer specific for each integration site. **c** The table summarizes the RMCE acceptor loci present and lists the gender of each F2 pig

A progeny of 27 healthy F2 piglets was produced by mating F1 pig #2772 with four non-transgenic gilts (Göttingen minipig). Southern blot analysis (Fig. 4a) was performed with genomic DNA from the 27 piglets digested with *SpeI* and probed with the same Neo^r^ probe, as used previously (e.g. Fig. 1), and the identification number of each piglet is indicated beneath each lane. The lanes marked 2772 represent genomic DNA from the F1 pig #2772, and the four RMCE loci are represented by the four bands designated A, B, C, and D according to the decreasing length of the bands (Fig. 4a). The banding patterns indicate that all four RMCE loci, present in F1 pig #2772, segregate independent of each other in the F2 progeny (Fig. 4a) which is consistent with the RMCE loci being unlinked. To confirm the presence or absence of each RMCE locus in the genome of each F2 piglet, we conducted PCR analyses using locus-specific primers (see “Materials and methods”) and found complete agreement between the Southern blot results and the PCR results (compare lanes in Fig. 4a, b). The segregation results, summarized in Fig. 4c, allowed us to measure the GFP expression of each of the loci B, C, and D while the expression from locus A had to be determined indirectly, as this locus was always present together with one or more of the other loci.

**Fig. 5:**
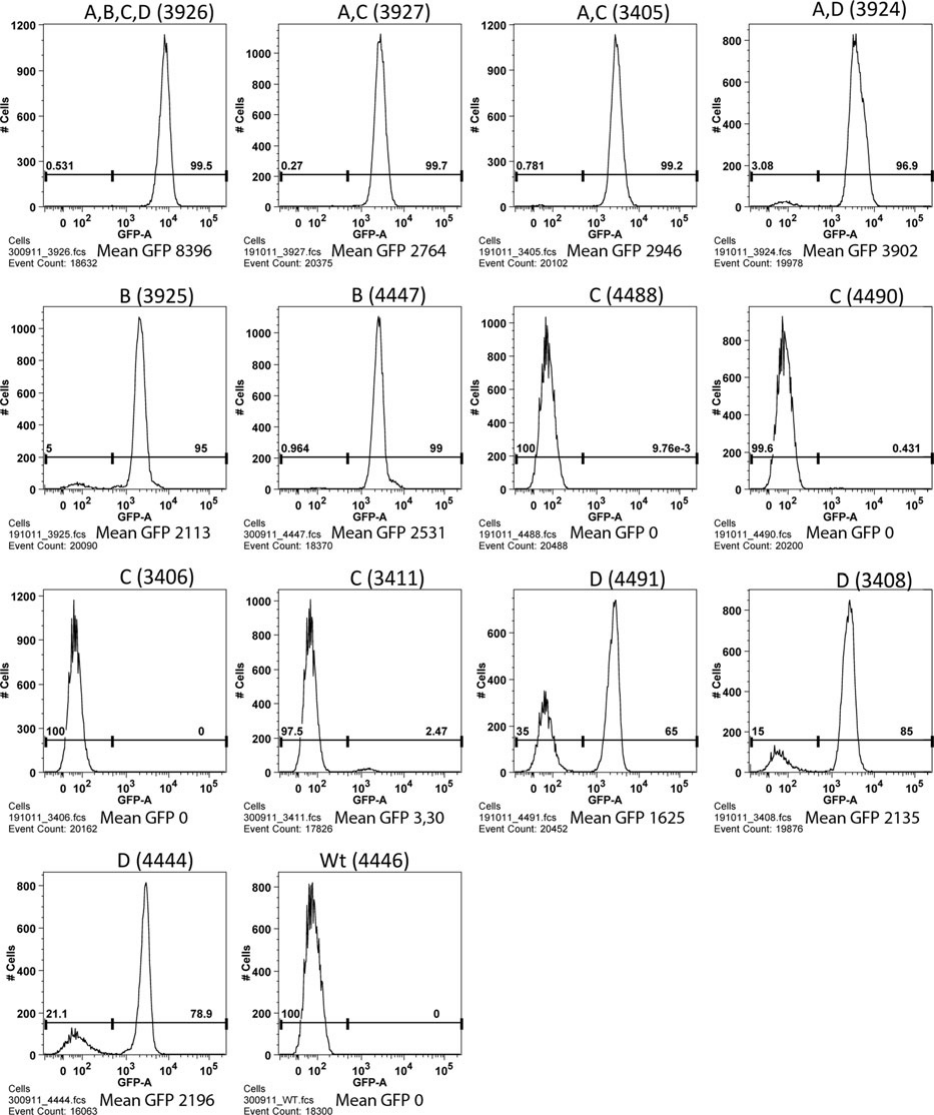
Flow cytometric GFP analysis of mononuclear cells isolated from blood samples of 13 F2 transgenic pigs with different RMCE acceptor loci. Each diagram represents a blood analysis from one pig. The number of the pig and the locus is listed on the top of the diagram. Around 20,000 cells were analyzed per sample. The mean GFP emission listed under each diagram

Fluorescence-activated cell sorting (FACS) of white blood cells indicated that RMCE locus C did not express GFP protein as the sorting pattern of cells from four different F2 piglets each carrying locus C as the only RMCE locus (piglets #4488, 4490, 3406, 3411) could not be distinguished from the sorting pattern of cells from piglet #4446 which carried no RMCE loci (Fig. 5). By contrast, the GFP activation intensity was at least one order of magnitude higher in cells from F2 piglets containing all four RMCE loci (piglet #3926), or locus A + C (piglets #3927, 3405), or locus A + D (piglet #3924), or locus B (piglets #3925, 4447), or locus D (piglets #4491, 3408, 4444). The apparent absence of GFP expression from locus C suggested that GFP expression detected in the piglets containing both the A and C loci originated mostly or exclusively from the A locus. Similar FACS results were obtained with primary fibroblasts from each piglet (data not shown). We also obtained measurements of GFP-activated radiance from the surface of brain, bladder, colon, and lung tissues recovered from animals having all four loci (pig #2772), locus A + C (piglet #3927), locus B (piglet #4447), locus C (piglet #4488), locus D (piglet #4491), and the radiance pattern was similar to the FACS results (Supplement Fig. S4b). The expression profile was also determined by a qPCR study of the same tissues from these animals (Supplement Fig. S4). In conclusion, very little or no expression was detected from locus C and the highest expression level from locus A, while locus B and D appeared to have somewhat similar levels of expression. No or almost no expression was detected from locus D in tissues from colon and lung.

**Fig. 6:**
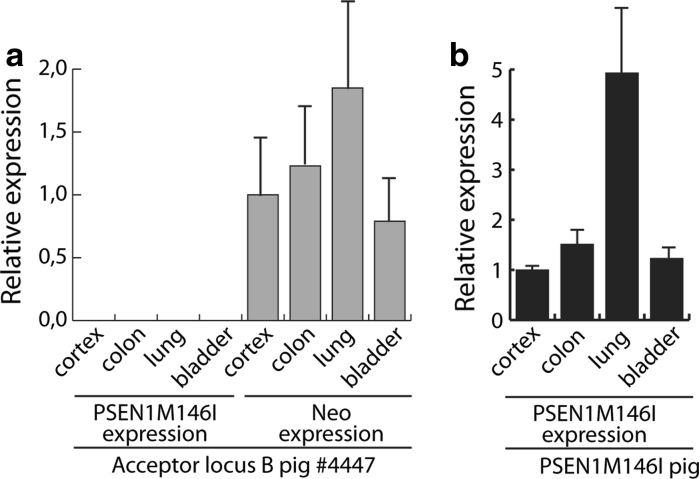
Expression pattern in four organs from two different transgenes located at acceptor locus B. Quantitative RT-PCR analysis of **a** the *Neo* and *PSEN1M146I* transgenes in pig 4447 or **b** the *PSEN1M146I* transgene in the *PSEN1M146I* pig. The expression of target mRNA was normalized to the expression of *HMBS*. All RNA extractions were performed in triplicates consisting of three separate extraction sites in the selected organ; cerebral cortex, colon, lung and bladder. Each qRT-PCR sample was run in technical triplicates. SD represented by *error bars* shows the variation between the three extraction sites within each organ. The expression level in cerebral cortex was normalized to the value of one

We next asked the question whether a RMCE event per se at a specific acceptor locus changes the expression from that locus. To this end we conducted a qPCR study of the expression profile of locus B in tissue specimens from cerebral cortex, colon, lung, and bladder before and after RMCE, i.e. comparing the activity of the UbiC promoter (before RMCE) with that of the enhanced UbiC promoter (after RMCE). We expected the two expression profiles to be similar if the RMCE event had no effect on the transcriptional activity of the locus. We used Neo^r^ qPCR primers [“Materials and methods”, and (Jakobsen et al. 2012)] to measure the mRNA levels generated by UbiC promoter. Figure 6a shows the mRNA levels in tissues originating from F2 piglet #4447 relative to the level in tissue from the cerebral cortex and normalized to the expression level of the housekeeping gene *HMBS*. The results indicate that locus B is transcriptionally active in these four tissues and 1.5–2 times more active in colon and lung tissues. qPCR was also done with PSEN1 primers specific of the human *PSEN1M146I* and, as expected, no mRNA was detected (Fig. 6a). These same primers was used in the qPCR study shown in Fig. 6b, where the activity of the enhanced UbiC promoter was measured in the same tissues but from one of the RMCE-generated piglets. The mRNA levels are shown relative to the level in tissue from the cerebral cortex and have been normalized to the expression level of *HMBS*. Although the expression appears to be 5 times higher in lung tissue the combined results from the tissues provide an expression profile similar to that observed before RMCE.

## Discussion

We generated pigs (F0) containing multiple SB transposon insertions using SB transposase-mediated transgenesis. These integrated transposons contain a cassette for RMCE. Through breeding we generated a F1 pig with four unlinked RMCE acceptor loci and subsequently bred F2 pigs with a single RMCE acceptor locus. By FACS analyses, GFP radiance measurements, and qPCR we show that of the four RMCE loci (A,B,C,D) three (A,B,D) are transcriptionally active in at least six different tissues, including cerebral cortex, while locus C is inactive in these tissues.

Recently, Garrels et al. verified the combination of the SB system and RMCE to produce RFP porcine fetuses from GFP fibroblasts with a single RMCE acceptor site (Garrels et al. 2011). Although we did not use microinjection of zygotes in the transposase-catalyzed transgenesis, our method is similar to that of Garrels et al. and confirms that it is possible to generate targeted transgenic pigs mediated by RMCE and SCNT. We here present RMCE piglets with one copy of the Alzheimer’s disease-causing mutation, *PSEN1M146I*, generated by targeting the acceptor loci in fibroblasts from the F1 pig using the Cre-loxP system in combination with minicircles and SCNT. At that time we did not know which RMCE locus to target, but we have recently obtained results showing that all four loci can be targeted (data not shown). Previous reports have described the ability of long-term Cre expression to introduce chromosomal aberration due to crossover between loxP sites (Collins et al. 2000). It was therefore of a concern whether it would be possible to produce viable pigs using RMCE in fibroblasts with four acceptor sites each containing 2 loxP sites even though Cre expression would only be transient. However, 21 piglets were born and the fraction of healthy piglet (16 with normal birth weight and no abnormalities) were slightly higher than our average for SCNT piglets.

Our RMCE-generated piglets are genetically identical to the donor pig (#2772) except for the replacement of the GFP gene with *PSEN1M146I* at one acceptor locus B, where it is actively transcribed (Figs. 3e, 6). Our Southern blot analyses did not show any random integration of *PSEN1M146I* in the genomes of RMCE piglets. Three cell colonies were pooled prior to SCNT but the Southern blots (Fig. 3b, c) indicate that all piglets originated from only one of the cell colonies, or, less likely, from two or all three colonies which would require that RMCE occurred more than once at only the RMCE acceptor locus B. In the F0 generation we generated three identical pigs produced from ten pooled colonies. We have described a similar event in a litter of five piglets produced from 80 pooled colonies and resulting in three different transgenic backgrounds (Jakobsen et al. 2011a). To explain the observation we suggest that (1) one of the cell colonies grows significantly faster than the rest of the colonies in the pool, or (2) cells from one of the colonies have a significantly better blastocyst-forming capacity, or (3) blastocysts made from some cells may be less viable and lost due to in utero selection. We pooled the cell colonies in order to reduce the number of passages needed to reach an acceptable number of cells for SCNT. It is our experience that single colonies grow poorly compared to pools and also that the rate of live-born transgenic pigs are higher when using pools compared to single colonies.

A site specific integration system, as the presented, has several advantages compared to random integration of the gene of interest (GOI)): it facilitates integration of an intact GOI into loci preselected for transcriptional activity; control of GOI copy number (up to four integrations in pig #2772); reduce the risk of interrupting porcine genes thereby increase the rate of healthy born transgenic pigs; avoid integration of prokaryotic DNA and antibiotic resistant genes from plasmid backbones. In addition the donor pig (#2772) may serve as a phenotypic control of the effect of the GOI in RMCE generated pigs as they have an identical genome except at the RMCE acceptor locus. We have previously used minicircles in Flp-in assays of various cell lines (Jakobsen et al. 2010) and now present the minicircle application in combination with RMCE. Using minicircles in RMCE removes the requirement for a negative selection marker as random integration of minicircles will separate the positive selection marker from the promoter. This will most likely disrupt expression of the positive selection marker killing non-RMCE cells. An alternative approach for minicircle production has been introduced recently in gene therapy studies of mice (Kay et al. 2010). This is to our knowledge, the first time a transgenic mammal has been generated using minicircles and SCNT.

The generation of F2 piglets with the single RMCE acceptor locus B gave us an opportunity to address whether RMCE per se interferes with expression from a transcriptionally active acceptor locus. We performed a thorough qPCR study of RMCE acceptor locus B before and after RMCE and found that the expression profiles composed of qPCR from four different tissues were similar.

We believe that transgenesis in a preselected transcriptionally active acceptor locus by RMCE and followed by SCNT represents a useful tool in the effort to develop large transgenic animals as human disease models.

## Materials and methods

### Statement of ethical approval

The pigs were housed and handled according to Danish law on genetically modified animals. The pigs were first anesthetized with a Zoletil mixture (10 ml Zoletil mixture: One bottle of Zoletil is dissolved in 2.5 ml torbugesic (10 mg/ml), 1.25 ml ketaminol (100 mg/ml) and 6.25 ml rompun (20 mg/ml)). After anesthetizing the pigs they were sacrificed by injecting Pentobarbital (300 mg/ml) directly into the heart. 1 ml of Pentobarbital was used per 3 kg of the pigs bodyweight. The experiments were conducted and approved by the Danish Animal Experiments Inspectorate (license no. 2006-561/1156 and 2009-561/1733).

### Vector construction

Construction of the pSBT/Ubi-GIN was described (Jakobsen et al. 2011a) Wt loxP was introduced just upstream of the Ubi C promoter using the two primers 5′-GGC TAC GCG TAT AAC TTC GTA TAA TGT ATG CTA TAC GAA GTT ATA GAT CTG GCC TCC GCG CCG G-3′ (loxP is underlined) and 5′-ATT AGC GAA GGC CTC AAG AC-3′ in a PCR performed with pSBT/Ubi-GIN as template. The fragment was inserted by *MluI*/*StuI*-digested pSBT/Ubi-GIN to create pSBT/loxP-Ubi-GIN. The mutated loxP, annotated loxP*, is named loxP257 and is described in Wong et al. (2005). LoxP257 was made by oligo annealing of the following oligos: 5′-CGC G ATA ACT TCG TAT AGG AGA CTT TAT ACG AAG TTA T-3′and 5′-CGC G ATA ACT TCG TAT AAA GTC TCC TAT ACG AAG TTA T-3′ (loxP257 is underlined). The double-stranded oligo was inserted into *AscI* site in pSBT/loxP-Ubi-GIN thereby creating pSBT/floxedUbi-GIN. Minicircles were produced using the protocol described (Jakobsen et al. 2010). Minicircles were isolated through a phenol–chloroform purification step to reduce contribution from the buffer used to create the minicircles. To create the templates for the minicircles, a wt loxP site and the CMV enhancer was inserted upstream of pSBT/Ubi-GIP described in (Jakobsen et al. 2011a) using the pcDNA5/FRT as template and the following primers: 5′-GGA TGA GCT CAT AAC TTC GTA TAA TGT ATG CTA TAC GAA GTT AT GAT GTA CGG GCC AGA TAT CA-3′ (underlined is loxP sequence) and 5′-GGT AAC GCG TAC CAT GGT AAT AGC GAT GAC-3′. The fragment was inserted by *SacI*/*MluI*-digested pSBT/Ubi-GIP to create pSBT/loxP-Ei-Ubi-GIP. loxP257 was inserted in pSBT/loxP-Ei-Ubi-GIP the same way as described for pSBT/loxP-Ubi-GIN, thereby creating pSBT/floxed-Ei-Ubi-GIP. The *NcoI* site upstream of GFP was changed to a unique *PacI* site through site-directed mutagenesis enabling removal of GFP and replacement of *PSEN1M146I* through *PacI*/*AgeI* digestion. *PSEN1M146I* was amplified from pPDGFβ-*PSEN1M146I* using the following primers: 5′-CGA TTT AAT TAA ATG ACA GAG TTA CCT GCA CCG-3′ and 5′-CCT AAC CGG TCT AGA TAT AAA ATT GAT GGA A-3′. *PSEN1M146I* minicircles were produced using the pSBT/floxed-Ei-Ubi-*PSEN1M146I* plasmid with the following primers: 5′-CGG CCA GTG AAT TCG AGC TC-3′ and 5′-C’GA TGA GCT CGA TAC ATT GAT GAG TTT GGA C-3′.

The PCR product was cleaved using the *SacI* restriction enzyme and subsequently ligated to create circular DNA, as described in Jakobsen et al. (2010).

### Transfection of fibroblasts to generate F0 pig

Fibroblasts were cultured from ear biopsies of newborn Göttingen minipig no. 74113 (Ellegaard Göttingen Minipigs A/S, Soroe Landevej 302, DK-4261 Dalmose, Denmark) as previously described (Kragh et al. 2009). The cells were grown in Dulbecco’s Modified Eagle Medium (DMEM) to 50 % confluence and passage for further expansion prior to freezing of aliquots. For production of a transgenic litter, 2 × 10^5^ fibroblasts were transfected in a six-well dish (in 2.5 ml medium) using 0.9 μg of the pSBT/floxedUbi-GIN transposon plasmid and 0.1 μg pCMV-HSB3 or as control 0.1 μg pUC19 plasmid. 3 μl FuGENE-6 was used in the reaction mixture. On the following day, the cells were washed with PBS and transferred to a 60-cm^2^ dish and subsequently cultured in G418-containing medium (0.75 μg/ml) for 2 weeks. A total of 10 colonies were pooled and grown for 9 days prior to SCNT by handmade cloning.

### Somatic cell nuclear transfer (SCNT) by handmade cloning

Handmade cloning was performed as previously described (Du et al. 2007; Kragh et al. 2009). Briefly, oocytes with partially digested zona pellucida were enucleated by oriented bisection according to the polar body position. For each oocyte, the part without chromatin, i.e. the cytoplast, was collected and electrofused with one transgenic fibroblast. Another cytoplast was then electrofused with each cytoplast-fibroblast pair during a second fusion round which also activated the reconstructed embryo. After 5 or 6 days of in vitro culture, morulae and blastocysts of excellent quality were selected for surgical transfer to Danish landrace sow on day 4 or 5 after heat, registered 5 days after weaning (Boyd 2005). Pregnancy in the surrogate sow was diagnosed by ultrasonography on day 28 and confirmed during later stages of the pregnancy. Pigs were delivered by natural birth after induction with prostaglandin on day 114 and raised by their surrogate sow. Pregnancy was established by transfer of 12 day six morulae and 72 transgenic blastocysts to the first surrogate sow and 45 day five morulae and 49 transgenic blastocysts to the second sow, which delivered 6 and 15 RMCE piglets, respectively. The experiments were conducted according to the Danish Animal Experiments Inspectorate (license no. 2006-561/1156 and 2009-561/1733).

### RMCE transfection in fibroblasts from F1 pig #2772

Neonatal fibroblasts from pig #2772 were grown to 90 % confluence in 75 cm^2^ flasks. The fibroblasts were harvested and suspended in 200 μl cold serum-free DMEM. 0.75 μg of *PSEN1M146I* minicircle and 15 μg of PGK-Cre plasmid or 15 μg pUC19 plasmid as a negative control were co-electroporated into 100 μl of fibroblasts. A 0.2 cm electrorode (gap 5) sterile and disposable cuvette was used in the gene pulser xcell electroporation system (Bio-Rad 617BR1). The program was set to a single pulse of 110 votage for 25.0 ms. The cells were subsequently seeded in a 60-cm^2^ dish and washed with PBS on the following day to remove dead cells (around 30 %). DMEM containing 1 μg/ml puromycin was added to the cells the following day. Selection continued for 5 days and afterwards the cells were reseeded directly in 2 μg/ml puromycin medium for additional 9 days. Puromycin selection medium was changed every third day. The cells were allowed to expand into sizable colonies for 3 days without puromycin before being harvested and transferred to 6-well dishes with 3 ml medium. Three to four days after, 50 % of the cells were stored at −135 °C frozen down and the remaining cells further expanded for a maximum of 12 days to obtain as many cells as possible for DNA/RNA extraction. The three cell colonies used for SCNT were thawed, pooled, and expanded for 10 days prior to SCNT.

### DNA/RNA extraction and cDNA synthesis

DNA and RNA were extracted using the AllPrep DNA/RNA mini kit (Qiagen-80204) according to manufacturer’s protocol. DNA and RNA were eluted in 100 μl and 50 μl nuclease free water, respectively. cDNA was synthesized using the iScript cDNA synthesis kit (Bio-Rad-170-8891) from 0.2 μg total RNA. The cDNA was diluted tenfold with redistilled water before use for PCR.

### PCR on DNA or cDNA

PCR was done according to standard protocols in a volume of 50 μl with Phusion-polymerase (Finnzymes). 100 ng of genomic DNA or 10 μl of cDNA were used as templates. The primers used to verify RMCE are depicted in Fig. 1c. The primer sequences are given in numerical order: 5′-GAG TCA ATT GGA GGT GTA CC-3′, 5′-GGG TGA ATT TTG GCT CAT TCC-3′, 5′-CAG GCA TGG ATG ACC TTA TAG-3′, 5′-GCT GTG GAC TAC ATT ACT GTT G-3′. The primers used on cDNA to check for *PSEN1M146I* and Cre expression were as follows: 5′-GTG TTC TGG TTG GTA AAG CCT C-3′ and 5′-GCT CGT AGA AGG GGA GGT TG-3′, 5′-CAT TTG GGC CAG CTA AAC AT-3′ and 5′-CCC GGC AAA ACA GGT AGT TA-3′. The following primer pairs were used to verify the RMCE in the produced piglet at acceptor locus B: Upstream LIR to GFP or PS1M146I: 5′-CCA TGG CAA TAC CAG ATT CC-3′, 5′-AGT TGT ACT CCA GCT TGT GC-3′, 5′-CAG GCA TGG ATG ACC TTA TAG-3′.

### Quantitative PCR

Twenty five nano grams genomic DNA or 1.25 μl cDNA were used as template to determine the Neo^r^ copy number or the relative GFP mRNA levels, respectively. The templates were mixed with 3.75 μl mastermix (containing 0.625 pmol of each primer and 2.5 μl SYBR GREEN (Roche-04887352001)) giving a total volume of 5 μl. The mixture was pipetted in each 384-well using a Beckman Coulter Biomek 3000 robot. Each reaction was performed in three wells to obtain a technical triplicate. The qPCR plate was given a short spin before being put into the iCycler Thermal Cycler (Bio-Rad). Cycle conditions were: 95 °C, 10 s; 60 °C, 20 s; 72 °C, 30 s; 40 repeats. The Neo^r^ cycle number was normalized to the endogenous *GLIS* 3 representing 2 copies. The levels of mRNA were normalized to the geometric mean of *HMBS* and quantified using the x_0_ method (Thomsen et al. 2010). The GLIS 3 primers were: 5′-GTT TGC ACC TTC TGC TCC AT-3′ and 5′-GAA AAG AAG AGC TTG TGT CTG G-3′. The Neo^r^ primers: 5′-TGCTCC TGCCGAGAAAGTAT-3′ and 5′-GCTCTTCGTCCAGATCATCC-3′. The GFP primers: 5′-GCA TCA AGG TGA ACT TCA GA-3′ and 5′-GAC TGG GTG CTC AGG TAG TG-3′. The HMBS reference primers are 5′-AGGATGGGCAACTCTACCTG-3′ and 5′-AGATGTTCTCAAACGCTTCG-3′ described in Nygard et al. (2007). The PSEN1M146I primers are 5′-TTAAAACCTATAACGTTGCTG-3′ and 5′-GCCTGCTGGAGTCGAAGTGGA-3′. On average, the qPCR cycle number using Neo^r^ primers was 0.5 lower compared to HMBS. For PSEN1M146I primers the cycle number was on average 4.5 lower compared to HMBS.

### Southern blotting

Southern blotting was carried out as described previously using the same stringency condition, the same isotope and Neo^r^ probe (Jakobsen et al. 2011a). In addition, an 800-bp fragment generated by *AclI* and *BsrGI* digestion of the *PSEN1M146I* transgene was used as a probe to reveal the presence of *PSEN1M146I* transgenes in the RMCE piglets.

### Long distance inverse (LDI)-PCR and analysis of GFP expression

LDI-PCR and analysis of GFP expressing organs have been described previously (Jakobsen et al. 2011a). The following primer pairs were used in the *BsrGI* LDI-PCR to reveal the transposon insertion site (TIS) in chromosome 7 and 14: 5′-CAT GTC TGG ATC CCA TCA CAA A-3′ and 5′-CTT GTG GAA GGC TAC TCG AA-3′ (Fig. 1a primer pair a), 5′-TAC GCT TGA GGA GAG CCA TT-3′ and 5′-GAG GAA CTG CTT CCT TCA CG-3′ (Fig. 1a primer pair b). The following primer pairs were used in the *SpeI* LDI-PCR to reveal the TIS in chromosome 1, * and 14: 5′-CAT GTC TGG ATC CCA TCA CAA A-3′ and 5′-CTT GTG GAA GGC TAC TCG AA-3′ (Fig. 1a primer pair a), 5′-AGT TGT ACT CCA GCT TGT GC-3′ and 5′-AAG TCG TGC TGC TTC ATG TG-3′ (Fig. 1a primer pair c). Confirmations of the genomic sites were performed with a GFP primer (5′-AGT TGT ACT CCA GCT TGT GC-3′) and a primer unique to the genomic site with the following sequences: TIS A: 5′-GAG CTA GGC CTG GGG ATA CT-3′; TIS B: 5′-CCA TGG CAA TAC CAG ATT CC-3′; TIS C: 5′-TCA TTC TTG TGC CTG TGG AC-3′; TIS D: 5′-TCC CAC TTC CCA TAC TCA GC-3′.

### Luminescence imaging

Every organ from all of the transgenetic pigs was imaged using the IVIS^®^ imaging system (Caliper Life Science, Belgium). The IVIS^®^ imaging system was set to detect GFP. Total photon emissions from predefined regions of interest were defined as a whole organ. The captured images were then quantified by using the Living Image software package (Caliper Life Science, Belgium). Organs from wt pigs were imaged to detect any background signals. The background signal from the wt pigs were used to normalize the fluorescence signal from the transgenetic pigs. Negative signal values can occur due to a higher background signal than the signal from the transgenetic pig.

### Flow cytometry of blood samples

Whole blood from pigs were collected from the neck vein in sodium citrate using Vacutainer™CPT™ tubes (BD). Mononuclear cells were separated by density gradient centrifugation through a polyester gel in the collection tube according to the manufacturers instructions. Cells were washed twice in PBS before resuspended in PBS containing 0.1 % BSA for flow cytometric analysis of GFP expression in all mononuclear cells. A FACSAriaIII (BD) using a 488 nm laser and a 530/30 nm bandpass filter was used for detection of GFP. FlowJo software (v.9.5.1, Tree Star Inc., Ashland, OR, USA) was used for analysis.

## Electronic supplementary material

Below is the link to the electronic supplementary material.

Supplementary material 1 (PDF 247 kb)

Supplementary material 2 (PDF 317 kb)

Supplementary material 3 (PDF 380 kb)

Supplementary material 4 (PDF 425 kb)

## References

[CR1] Boyd J (2005). Mouse models of gynecologic pathology. N Engl J Med.

[CR2] Brevini TA, Pennarossa G, Attanasio L, Vanelli A, Gasparrini B, Gandolfi F (2010). Culture conditions and signalling networks promoting the establishment of cell lines from parthenogenetic and biparental pig embryos. Stem Cell Rev.

[CR3] Brevini TA, Pennarossa G, Gandolfi F (2010). No shortcuts to pig embryonic stem cells. Theriogenology.

[CR4] Capecchi MR (1989). Altering the genome by homologous recombination. Science.

[CR5] Carlson CM, Dupuy AJ, Fritz S, Roberg-Perez KJ, Fletcher CF, Largaespada DA (2003). Transposon mutagenesis of the mouse germline. Genetics.

[CR6] Carlson CM, Frandsen JL, Kirchhof N, McIvor RS, Largaespada DA (2005). Somatic integration of an oncogene-harboring Sleeping Beauty transposon models liver tumor development in the mouse. Proc Natl Acad Sci USA.

[CR7] Carlson DF, Garbe JR, Tan W, Martin MJ, Dobrinsky JR, Hackett PB, Clark KJ, Fahrenkrug SC (2011). Strategies for selection marker-free swine transgenesis using the Sleeping Beauty transposon system. Transgenic Res.

[CR8] Carlson DF, Geurts AM, Garbe JR, Park CW, Rangel-Filho A, O’Grady SM, Jacob HJ, Steer CJ, Largaespada DA, Fahrenkrug SC (2011). Efficient mammalian germline transgenesis by cis-enhanced Sleeping Beauty transposition. Transgenic Res.

[CR9] Clark KJ, Carlson DF, Foster LK, Kong BW, Foster DN, Fahrenkrug SC (2007). Enzymatic engineering of the porcine genome with transposons and recombinases. BMC Biotechnol.

[CR10] Cobellis G, Nicolaus G, Iovino M, Romito A, Marra E, Barbarisi M, Sardiello M, Di Giorgio FP, Iovino N, Zollo M, Ballabio A, Cortese R (2005). Tagging genes with cassette-exchange sites. Nucleic Acids Res.

[CR11] Collins EC, Pannell R, Simpson EM, Forster A, Rabbitts TH (2000). Inter-chromosomal recombination of Mll and Af9 genes mediated by cre-loxP in mouse development. EMBO Rep.

[CR12] Doetschman T, Gregg RG, Maeda N, Hooper ML, Melton DW, Thompson S, Smithies O (1987). Targetted correction of a mutant HPRT gene in mouse embryonic stem cells. Nature.

[CR13] Du Y, Kragh PM, Zhang Y, Li J, Schmidt M, Bogh IB, Zhang X, Purup S, Jorgensen AL, Pedersen AM, Villemoes K, Yang H, Bolund L, Vajta G (2007). Piglets born from handmade cloning, an innovative cloning method without micromanipulation. Theriogenology.

[CR14] Dupuy AJ, Fritz S, Largaespada DA (2001). Transposition and gene disruption in the male germline of the mouse. Genesis.

[CR15] Dupuy AJ, Akagi K, Largaespada DA, Copeland NG, Jenkins NA (2005). Mammalian mutagenesis using a highly mobile somatic Sleeping Beauty transposon system. Nature.

[CR16] Garrels W, Mates L, Holler S, Dalda A, Taylor U, Petersen B, Niemann H, Izsvak Z, Ivics Z, Kues WA (2011). Germline transgenic pigs by Sleeping Beauty transposition in porcine zygotes and targeted integration in the pig genome. PLoS ONE.

[CR17] Geurts AM, Collier LS, Geurts JL, Oseth LL, Bell ML, Mu D, Lucito R, Godbout SA, Green LE, Lowe SW, Hirsch BA, Leinwand LA, Largaespada DA (2006). Gene mutations and genomic rearrangements in the mouse as a result of transposon mobilization from chromosomal concatemers. PLoS Genet.

[CR18] Jakobsen J, Mikkelsen J, Nielsen A (2010). Elimination of the plasmid bacterial backbone in site-directed transgenesis. Biotechniques.

[CR19] Jakobsen JE, Li J, Kragh PM, Moldt B, Lin L, Liu Y, Schmidt M, Winther KD, Schyth BD, Holm IE, Vajta G, Bolund L, Callesen H, Jorgensen AL, Nielsen AL, Mikkelsen JG (2011). Pig transgenesis by Sleeping Beauty DNA transposition. Transgenic Res.

[CR20] Jakobsen JE, Li J, Moldt B, Kragh PM, Callesen H, Hertz JM, Bolund L, Jorgensen AL, Mikkelsen JG, Nielsen AL (2011). Establishment of a pig fibroblast-derived cell line for locus-directed transgene expression in cell cultures and blastocysts. Mol Biol Rep.

[CR21] Jakobsen JE, Dantoft TM, Johansen MG, Jorgensen AL (2012). Expression pattern of a single transgene cassette located in endogenous GLIS3 of cloned pigs; a nested situation. Gene.

[CR22] Kay MA, He CY, Chen ZY (2010). A robust system for production of minicircle DNA vectors. Nat Biotechnol.

[CR23] Kitada K, Ishishita S, Tosaka K, Takahashi R, Ueda M, Keng VW, Horie K, Takeda J (2007). Transposon-tagged mutagenesis in the rat. Nat Methods.

[CR24] Kragh PM, Nielsen AL, Li J, Du Y, Lin L, Schmidt M, Bogh IB, Holm IE, Jakobsen JE, Johansen MG, Purup S, Bolund L, Vajta G, Jorgensen AL (2009). Hemizygous minipigs produced by random gene insertion and handmade cloning express the Alzheimer’s disease-causing dominant mutation APPsw. Transgenic Res.

[CR25] Mates L, Chuah MK, Belay E, Jerchow B, Manoj N, Acosta-Sanchez A, Grzela DP, Schmitt A, Becker K, Matrai J, Ma L, Samara-Kuko E, Gysemans C, Pryputniewicz D, Miskey C, Fletcher B, VandenDriessche T, Ivics Z, Izsvak Z (2009). Molecular evolution of a novel hyperactive Sleeping Beauty transposase enables robust stable gene transfer in vertebrates. Nat Genet.

[CR26] Nygard AB, Jorgensen CB, Cirera S, Fredholm M (2007). Selection of reference genes for gene expression studies in pig tissues using SYBR green qPCR. BMC Mol Biol.

[CR27] Ostedgaard LS, Meyerholz DK, Chen JH, Pezzulo AA, Karp PH, Rokhlina T, Ernst SE, Hanfland RA, Reznikov LR, Ludwig PS, Rogan MP, Davis GJ, Dohrn CL, Wohlford-Lenane C, Taft PJ, Rector MV, Hornick E, Nassar BS, Samuel M, Zhang Y, Richter SS, Uc A, Shilyansky J, Prather RS, McCray PB Jr, Zabner J, Welsh MJ, Stoltz DA (2011) The deltaF508 mutation causes CFTR misprocessing and cystic fibrosis-like disease in pigs. Sci Transl Med 3(74):74ra24. doi:10.1126/scitranslmed.3001868 10.1126/scitranslmed.3001868 PMC3119077 21411740

[CR28] Osterwalder M, Galli A, Rosen B, Skarnes WC, Zeller R, Lopez-Rios J (2010). Dual RMCE for efficient re-engineering of mouse mutant alleles. Nat Methods.

[CR29] Rogers CS, Hao Y, Rokhlina T, Samuel M, Stoltz DA, Li Y, Petroff E, Vermeer DW, Kabel AC, Yan Z, Spate L, Wax D, Murphy CN, Rieke A, Whitworth K, Linville ML, Korte SW, Engelhardt JF, Welsh MJ, Prather RS (2008). Production of CFTR-null and CFTR-deltaF508 heterozygous pigs by adeno-associated virus-mediated gene targeting and somatic cell nuclear transfer. J Clin Invest.

[CR30] Rogers CS, Stoltz DA, Meyerholz DK, Ostedgaard LS, Rokhlina T, Taft PJ, Rogan MP, Pezzulo AA, Karp PH, Itani OA, Kabel AC, Wohlford-Lenane CL, Davis GJ, Hanfland RA, Smith TL, Samuel M, Wax D, Murphy CN, Rieke A, Whitworth K, Uc A, Starner TD, Brogden KA, Shilyansky J, McCray PB, Zabner J, Prather RS, Welsh MJ (2008). Disruption of the CFTR gene produces a model of cystic fibrosis in newborn pigs. Science.

[CR31] Schebelle L, Wolf C, Stribl C, Javaheri T, Schnutgen F, Ettinger A, Ivics Z, Hansen J, Ruiz P, von Melchner H, Wurst W, Floss T (2010). Efficient conditional and promoter-specific in vivo expression of cDNAs of choice by taking advantage of recombinase-mediated cassette exchange using FlEx gene traps. Nucleic Acids Res.

[CR32] Schmidt M, Kragh PM, Li J, Du Y, Lin L, Liu Y, Bogh IB, Winther KD, Vajta G, Callesen H (2010). Pregnancies and piglets from large white sow recipients after two transfer methods of cloned and transgenic embryos of different pig breeds. Theriogenology.

[CR33] Schmidt M, Winter KD, Dantzer V, Li J, Kragh PM, Du Y, Lin L, Liu Y, Vajta G, Sangild PT, Callesen H, Agerholm JS (2011). Maternal endometrial oedema may increase perinatal mortality of cloned and transgenic piglets. Reprod Fertil Dev.

[CR34] Schnutgen F, De-Zolt S, Van Sloun P, Hollatz M, Floss T, Hansen J, Altschmied J, Seisenberger C, Ghyselinck NB, Ruiz P, Chambon P, Wurst W, von Melchner H (2005). Genomewide production of multipurpose alleles for the functional analysis of the mouse genome. Proc Natl Acad Sci USA.

[CR35] Thompson S, Clarke AR, Pow AM, Hooper ML, Melton DW (1989). Germ line transmission and expression of a corrected HPRT gene produced by gene targeting in embryonic stem cells. Cell.

[CR36] Thomsen R, Solvsten CA, Linnet TE, Blechingberg J, Nielsen AL (2010). Analysis of qPCR data by converting exponentially related Ct values into linearly related X0 values. J Bioinform Comput Biol.

[CR37] Whyte JJ, Prather RS (2011). Genetic modifications of pigs for medicine and agriculture. Mol Reprod Dev.

[CR38] Wong ET, Kolman JL, Li YC, Mesner LD, Hillen W, Berens C, Wahl GM (2005). Reproducible doxycycline-inducible transgene expression at specific loci generated by Cre-recombinase mediated cassette exchange. Nucleic Acids Res.

