# Supplementary material for: Generation of minipigs with targeted transgene insertion by recombinase-mediated cassette exchange (RMCE) and somatic cell nuclear transfer (SCNT)
Source: Transgenic Res. 2012 Oct 31;22(4):709–23. doi: 10.1007/s11248-012-9671-6 (PMC3712138; doi:10.1007/s11248-012-9671-6)
Supplement: Supplementary file 1 — Supplementary material 1 (PDF 247 kb) [file 11248_2012_9671_MOESM1_ESM.pdf]

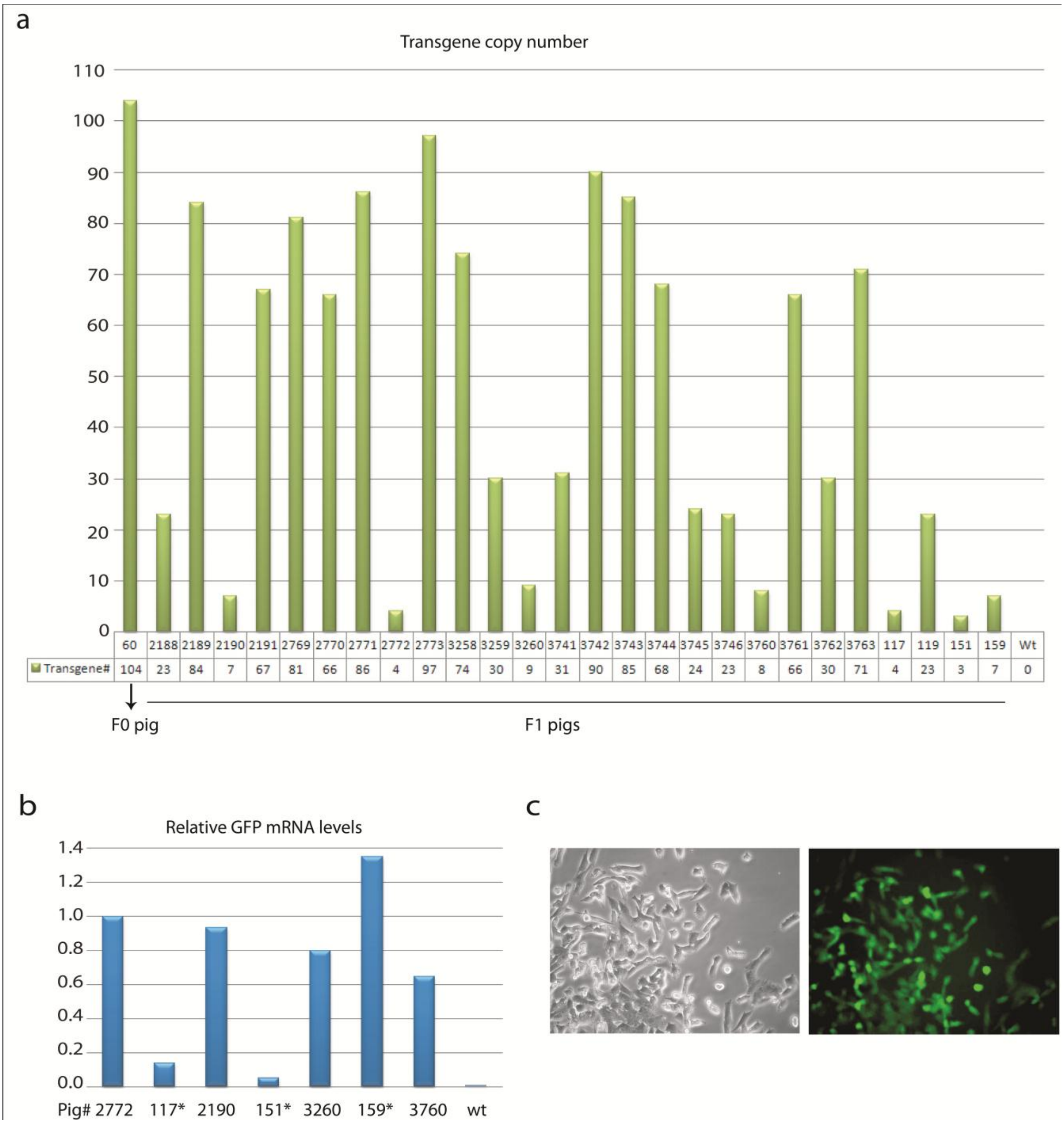

Fig. S1. Identification of a pig with few active acceptor loci. **a.** Twenty-six F1 pigs were generated from a male pig (F0, #60) with multiple SBT/floxedUbi-GIN insertions. The transgene copy number in #60 and its F1 progeny was determined by qPCR on genomic DNA using primers amplifying part of *Neo*^{r}. The individual pig number is written below each column and beneath that the estimated transgene copy number. **b.** Seven of the twenty-six F1 pigs with transgene copy numbers less than 10 were analyzed for GFP mRNA expression by RT-qPCR on cDNA made from RNA isolated from fibroblasts. The mRNA expression level observed for pig #2772 was normalized to the value of one. Pigs with number marked with * died within a couple of weeks after birth. **c.** Fibroblasts from pig #2772 analyzed by epifluorescence microscopy to reveal GFP expression. *Left side*, fibroblasts viewed under normal light. *Right side*, fibroblasts viewed under blue light.
