# Supplementary material for: Generation of minipigs with targeted transgene insertion by recombinase-mediated cassette exchange (RMCE) and somatic cell nuclear transfer (SCNT)
Source: Transgenic Res. 2012 Oct 31;22(4):709–23. doi: 10.1007/s11248-012-9671-6 (PMC3712138; doi:10.1007/s11248-012-9671-6)
Supplement: Supplementary file 2 — Supplementary material 2 (PDF 317 kb) [file 11248_2012_9671_MOESM2_ESM.pdf]

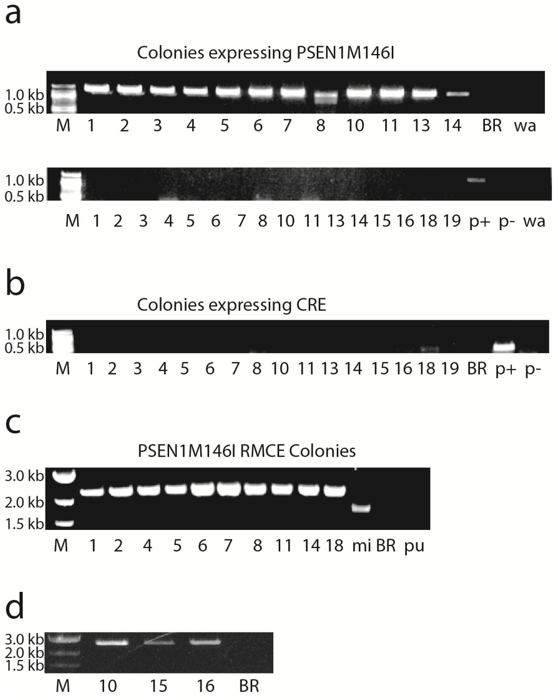

Fig. S2. Identification of RMCE colonies which express *PSEN1M146I* but not Cre recombinase. DNA and cDNA from colonies are used as template to evaluate if RMCE had taken place and if fibroblasts from the colony express *PSEN1M146I* and Cre. **a** *Top panel:* PCR on cDNA made from total RNA isolated from fibroblasts of various colonies. M is a 0.1 kb ladder. The number beneath each lane identifies each colony. BR represents cDNA from fibroblasts before RMCE was carried out. wa represents a water control. *Lower panel:* PCR with the same primers and conditions was carried out on colony fibroblasts RNA but no reverse transcriptase (RT) was added to the cDNA synthesis. p+ indicates a positive sample. p- indicates the –RT sample of p+. **b** Similar to **a** except that the cDNA generated from RNA extracted from the colonies was used for PCR analysis to reveal whether or not Cre was expressed in the individual colonies. **c** DNA from various colonies was used for PCR with primer combination 1+3, shown in Figure 1c, to reveal if RMCE had taken place for *PSEN1M146I* minicircles. M is a 1 kb ladder. “mi” represents *PSEN1M146I* minicircles. BR represents DNA from fibroblasts before RMCE was carried out. “pu” represents DNA isolated from fibroblasts co-tranfected with minicircles and with pUC19 plasmid instead of Cre plasmid. **d** PCR amplicons using primer combination 2+4 (Fig. 1b) confirms that a complete RMCE had occurred for *PSEN1M146I* minicircles in colony 10, 15, and 16.
