# Supplementary material for: Generation of minipigs with targeted transgene insertion by recombinase-mediated cassette exchange (RMCE) and somatic cell nuclear transfer (SCNT)
Source: Transgenic Res. 2012 Oct 31;22(4):709–23. doi: 10.1007/s11248-012-9671-6 (PMC3712138; doi:10.1007/s11248-012-9671-6)
Supplement: Supplementary file 3 — Supplementary material 3 (PDF 380 kb) [file 11248_2012_9671_MOESM3_ESM.pdf]

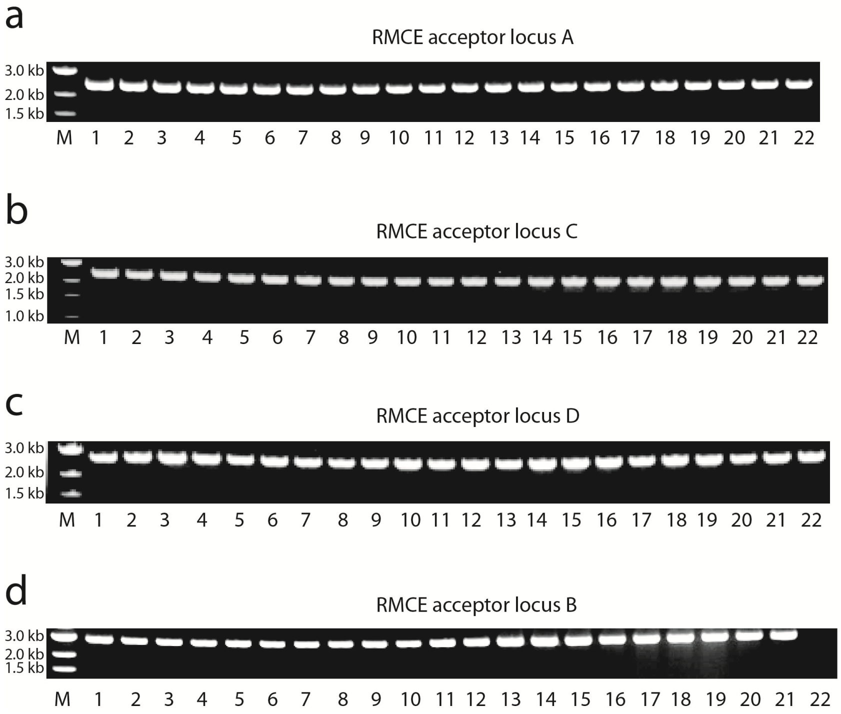


**Fig. S3.** Targeted and untargeted RMCE acceptor loci. M is a 1kb ladder. Lanes 1-21 represent PCR on DNA from all the RMCE piglets and lane 22 represents pig #2772. **a-c.** PCR using a genomic primer specific for each locus and a GFP primer on DNA from all the RMCE piglets and pig #2772 were carried out to investigate whether or not the gene cassette in the four acceptor loci was identical and positioned at the same genomic sites among all the RMCE piglets and pig #2772. **d.** A PCR using a genomic primer specific for locus B and a *PSEN1M146I* primer revealing that all the RMCE piglets were targeted at the same acceptor site, positioned at RMCE locus B.
