# Supplementary material for: Generation of minipigs with targeted transgene insertion by recombinase-mediated cassette exchange (RMCE) and somatic cell nuclear transfer (SCNT)
Source: Transgenic Res. 2012 Oct 31;22(4):709–23. doi: 10.1007/s11248-012-9671-6 (PMC3712138; doi:10.1007/s11248-012-9671-6)
Supplement: Supplementary file 4 — Supplementary material 4 (PDF 425 kb) [file 11248_2012_9671_MOESM4_ESM.pdf]

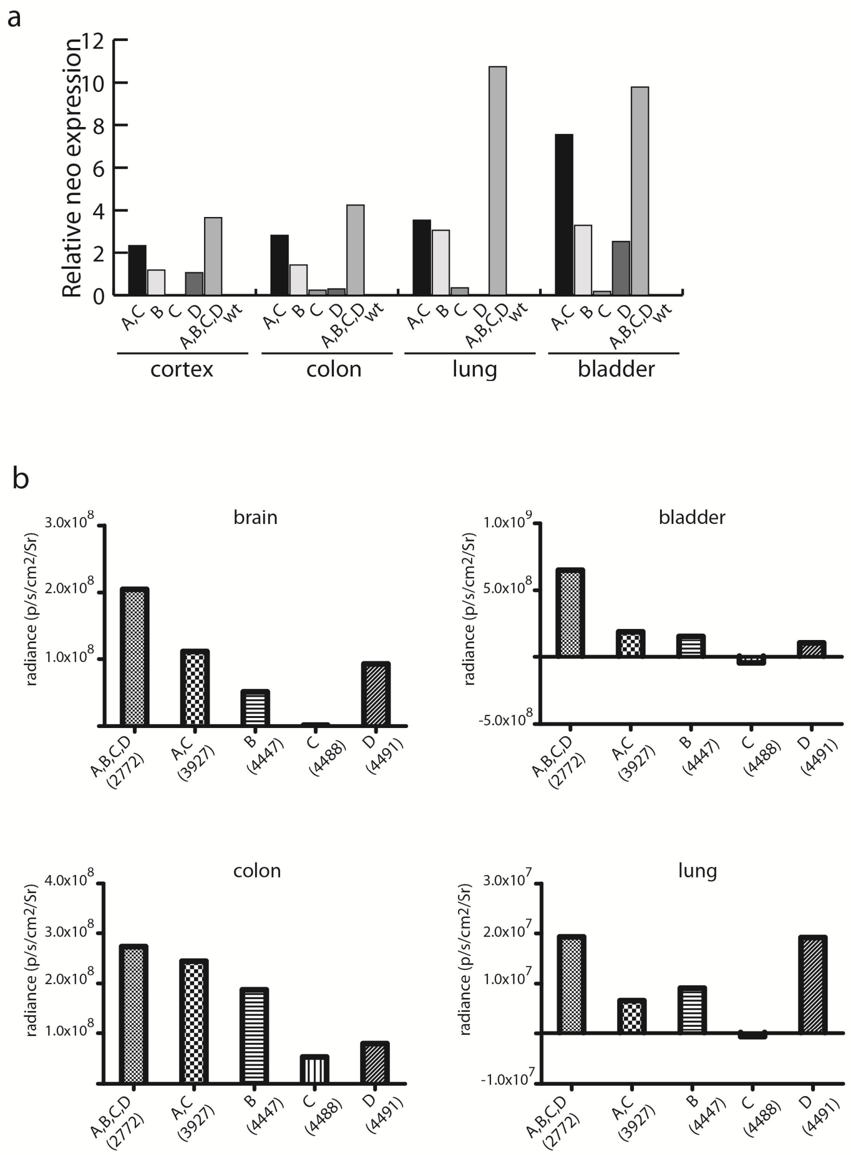

Fig. S4. Expression pattern in four organs from pigs with individual RMCE acceptor locus.

a. Quantitative RT- PCR analysis of the *GFP*-IRES-*Neo* bicistron at each of the four RMCE acceptor loci A, B, C and D. The expression of target mRNA was normalized to the expression of *HMBS*. All RNA extractions were performed once in the selected organ; cerebral cortex, colon, lung and bladder. Each qRT-PCR sample was run in technical triplicates. The expression level in cerebral cortex from locus B was normalized to the value of one. b**.** The same organs as in **a** were analyzed for GFP expression using the luminescent macroscopic imaging system (IVIS). The whole organ was scanned and the total photon emission was recorded and presented as photons per second per centimeter squared per steradian (p/s/cm2/sr). Background signal from a wt pig was subtracted from the presented value. The number of the pig and the acceptor locus is listed below each column.
